# Barriers to Implementing Intermittent Catheterisation in Spinal Cord Injury Patients in Northwest Regional Spinal Injuries Centre, Southport, U.K.

**DOI:** 10.1100/tsw.2011.12

**Published:** 2011-01-05

**Authors:** Subramanian Vaidyanathan, Bakul M. Soni, Gurpreet Singh, Tun Oo, Peter L. Hughes

**Affiliations:** ^1^ Regional Spinal Injuries Centre, District General Hospital, Southport, Merseyside, UK; ^2^ Department of Urology, District General Hospital, Southport, Merseyside, UK; ^3^ Department of Radiology, District General Hospital, Southport, Merseyside, UK

**Keywords:** spinal cord injury, intermittent catheterisation, tetraplegia, paraplegia, neurogenic bladder

## Abstract

Intermittent catheterisation is the preferred method of managing the neurogenic bladder in patients with spinal cord injury. However, spinal cord physicians experienced problems when trying to implement an intermittent catheterisation regime in some spinal cord injury patients in the northwest of England. We present illustrative cases to describe practical difficulties encountered by patients while trying to adopt an intermittent catheterisation regime. Barriers to intermittent catheterisation are (1) caregivers or nurses are not available to carry out five or six catheterisations a day; (2) lack of time to perform intermittent catheterisations; (3) unavailability of suitable toilet facilities in public places, including restaurants and offices; (4) redundant prepuce in a male patient, which prevents ready access to urethral meatus; (5) urethral false passage; (6) urethral sphincter spasm requiring the use of flexible-tip catheters and α-drenoceptor–blocking drugs; (7) reluctance to perform intermittent catheterisation in patients >60 years by some health professionals; and (8) difficulty in accessing the urethral meatus for catheterisation while the patient is sitting up, especially in female patients. These cases demonstrate the urgent need for provision of trained caregivers who can perform intermittent catheterisation, and improvement in public facilities that are suitable for performing catheterisation in spinal cord injury patients. Further, vigilance should be exercised during each catheterisation in order to prevent complications, such as urethral trauma and consequent false passages. Health professionals should make additional efforts to implement intermittent catheterisation in female spinal cord injury patients and in those >60 years.

## INTRODUCTION

Quite often, doctors take pride in writing about rare cases or discussing diagnostic puzzles. However, common problems, which continue to bother many patients, may not receive priority by doctors when publishing and sharing their experience with the medical fraternity. As an example of a day-to-day problem, which has not been solved even in the year 2010, we discuss barriers to implementing intermittent catheterisation in spinal cord injury patients in the northwest of England. This report of eight spinal cord injury patients illustrates the importance of medical as well as social aspects when implementing an intermittent catheterisation regime. Such an approach to medical care is best exemplified in relation to the control of neglected tropical diseases. Jerry Spiegel and colleagues, from the University of British Columbia, feel that there has been too much focus on the biomedical mechanisms and drug development for neglected tropical diseases, at the expense of attention to the social determinants of disease, a view shared by Burton Singer who argues that this represents another example of the inappropriate “overmedicalisation” of contemporary tropical disease control[1]. We believe that publication of this case series will help to highlight the importance of global care for spinal cord injury patients.

## CASE PRESENTATIONS

Patients with spinal cord injury who agreed to manage their bladder by intermittent catheterisation, but were unable to perform, were reviewed to find out the practical difficulties they face in carrying out intermittent catheterisations. Some examples are:

- Are these spinal cord injury patients unable to execute intermittent catheterisations because of lack of time?
- Are caregivers not available to do catheterisation five to six times a day?
- Is nonavailability of suitable toilet facilities in public places and shopping complexes a precluding factor for practising an intermittent catheterisation regime?
- Do spinal cord injury patients need to be placed on a bed for catheterisation and, therefore, are they unable to have intermittent catheterisations when they go out?
- Is there any mechanical hindrance to inserting a catheter per urethra?

### Case 1 – Lack of Time to Perform Intermittent Catheterisations

A British Caucasian male, born in 1969, sustained complete tetraplegia at the C-4 level in 1988 while playing rugby. He had been managing his bladder by sheath drainage. This patient had extremely devoted parents, and his family was a source of much inspiration and support. His brother designed a sophisticated computer-based environmental control system specifically for him. He was totally dependent on caregivers for all aspects of daily life. Intravenous urography, performed in 2003, showed normal kidneys, ureters, and bladder. In June 2010, this patient attended the spinal unit with a history of passing blood in his urine. Cytology of the urine showed large numbers of neutrophils and a few epithelial cells. No malignant cells were identified. Intravenous urography showed fullness of both renal pelvis and ureters. Ureters were tortuous; the urinary bladder was thick walled. Urine culture yielded growth of coliform species and *Pseudomonas aeruginosa*, both sensitive to gentamicin. Cystoscopy revealed a large volume of urine in the bladder with lots of debris. There was no tumour or vesical calculus. This patient was advised intermittent catheterisations at least four times a day. This patient’s wife was taught how to perform intermittent catheterisations. However, this patient was concerned that four catheterisations a day would be taking considerable time for his wife, who was already very busy with household chores and looking after their children. It required a lot of persuasion to convince the patient that the hassle of getting four catheterisations a day was worth the time spent on it. At present, this patient is managing to have four catheterisations a day, but continuing an intermittent catheterisation regime on a long-term basis might prove to be difficult.

### *Patient's Perspective*

"My wife has no time. She has to take the children to school. In the morning, catheterisation is a hassle. When I go out, I cannot have catheterisation. There is no suitable facility. When I play Bridge, I cannot have catheterisations done." This patient was requested to have a catheterisation just before an outing, thus giving him 4–6 h of freedom. This patient was provided with information about implementing intermittent catheterisation with a contained system of catheter attached to a collection bag, which required less space (LoFric Hydro-Kit).

## Case 2 – A Ventilator-Dependent Tetraplegic Patient was Not Happy with Intermittent Catheterisations, as He Could Not Go Out

A British Caucasian male, born in 1963, suffered multiple injuries following a motorbike accident in 2007, with a cardiac arrest at the scene. He sustained C1-C2 subluxation with complete C-0 tetraplegia of Frankel grade A. This patient had been managing a neurogenic bladder by a long-term indwelling urethral catheter. He developed orchitis in 2007 and 50 ml of pus was drained. In 2010, caregivers noticed a lot of sediment in his urine. There was recurrent blockage of the catheter and the patient developed urine infections. X-ray of the abdomen revealed opaque calculi in the urinary bladder. Urine microbiology revealed *Klebsiella pneumoniae*, *Enterococcus faecalis*, and *Pseudomonas aeruginosa*; meropenem was the only antibiotic to which all three organisms were sensitive.

The spinal cord physician discussed management of the neurogenic bladder with the patient and his team of 24-h caregivers. This patient was prescribed oxybutynin elixir through the gastrostomy and caregivers were taught the technique of intermittent catheterisation. However, the penile sheath would not stay in position. This patient was wearing a Conveen Optima 30-mm penile sheath. He was advised to try a P-sure sheath instead. Caregivers were taught how to apply the P-sure sheath over the penis. Another problem was this patient's inability to retain urine in his bladder. It was discovered that the patient's caregivers were giving 10 ml of oxybutynin elixir once a day at 0800 h. Oxybutynin elixir 5 ml contains 2.5 mg oxybutynin. Therefore, 10 ml would contain only 5 mg of oxybutynin. Five milligrams of oxybutynin once a day is not adequate for an adult patient. This patient's caregivers were advised to give oxybutynin elixir 5 mg four times a day through gastrostomy. After implementing the above changes, this patient was able to retain a penile sheath and caregivers were performing four hourly catheterisations. However, this patient was not happy to continue with intermittent catheterisations because he could not go out. Toilet facilities were not suitable in most public places for performing urethral catheterisation on a ventilator-dependent tetraplegic patient by caregivers. There was not enough space in almost all toilets to accommodate a tetraplegic patient seated in a large wheelchair to which a ventilator had been fitted and two caregivers simultaneously. The caregivers would have to bend forwards to undress the tetraplegic patient and perform urethral catheterisation, which proved almost impossible in public toilets. Therefore, this patient would have to rush back to his house from a shopping centre, restaurant, or a bar in order to undergo intermittent catheterisation. Even implementing intermittent catheterisation with a contained system of catheter attached to a collection bag (LoFric Hydro-Kit), which required less space, was not feasible in many public toilets. This patient felt that intermittent catheterisations were detrimental to his life style, which he wished to enjoy. This patient chose to have an indwelling urinary catheter, which enabled him to go to a bar and enjoy his drinks without worrying about intermittent catheterisations. Spinal cord physicians were unable to solve the problems faced by this patient in adopting an intermittent catheterisation regime.

## Case 3 – Lack of Caregivers to Perform Intermittent Catheterisation in a Tetraplegic Male Patient

A British Caucasian male, born in 1973, dove into the shallow end of the school's swimming pool in 1988; he hit the bottom of the pool and was unable to move his arms and legs. This young boy developed C-6 complete tetraplegia. X-ray of the cervical spine showed crush fractures of bodies of the fifth and sixth cervical vertebrae with anterior wedging; there was bilateral facetal dislocation of C-3/C-4. Gardener-Wells tongs were inserted; traction was applied with 7 lb. Subsequently, this patient underwent rehabilitation. As regards his urinary bladder, this patient was prescribed phenoxybenzamine 10 mg, three times a day, and was wearing a penile sheath.

In 2010, ultrasound examination of the urinary tract revealed normal echotexture of the left kidney, which measured 11.2 cm with good corticomedullary differentiation and cortical depth. The right kidney measured 10.5 cm with good corticomedullary differentiation and cortical depth. The urinary bladder was trabeculated with multiple diverticulae. There was no sonographic evidence of large bladder calculus. The patient was advised intermittent catheterisations at least four times a day, but the patient said that he was unable to have four catheterisations a day, as he required caregivers to perform the catheterisations.

### *Patient's Perspective*

"The reason for not being able to do four catheters a day is because I cannot do them myself. I need to have help, but I do not have assistance with me all day. I work full time and I have busy days and when I am at work in the day, I have no help from caregivers to do an intermittent. I do not always come home after work. Sometimes I work late or go out, so it is difficult to do one at say, 7 PM when I come home. My days are busy and full."

### *Comments*

Reconstructive hand surgery has been shown to improve the ability of a tetraplegic patient to perform self-catheterisation. Fifty-one percent (22/43) of the patients with a C-6 level of tetraplegia and 86% (12/14) of those with a C-7 or C-8 neurological level of injury could perform clean intermittent self-catheterisation independently following reconstructive hand surgery[2]. This patient had undergone reconstructive upper limb surgery, but he was still unable to perform intermittent catheterisation independently.

Assistive devices have been developed to enable spinal cord injury patients and traumatic brain injury patients with hemiplegia to perform intermittent catheterisation independently. The HouseHold device, developed by Dr. House, is basically a semi-rigid sleeve of fabric with a Velcro® fastener[3]. It is wrapped conically around the shaft of the penis to hold the penis in place so both of the patient's hands are free to help guide the catheter during its insertion when performing intermittent catheterisation. The HouseHold device offers an easily learned means for intermittent self-catheterisation in a subgroup of persons with tetraplegia who otherwise would require an alternative method of neurogenic bladder management. Carver described a penile prop, which was curved to fit the shape of the penis for stabilisation, angled to point the end of the penis upward for access to the urethra, and equipped with supporting legs to maintain equipment position[4]. It was fabricated of low-temperature thermoplastic material of 1/8-in. thickness. The penile prop was simple to fabricate and can be used to address any condition in which a patient may have difficulty maintaining penis position for self-catheterisation. Sadly, we did not have access to either of these devices, which could have enabled some spinal cord injury patients to perform intermittent catheterisation independently.

## Case 4 – Redundant Prepuce Preventing Easy Access to External Urethral Meatus in a Tetraplegic Patient

A British Caucasian male, born in 1971, sustained C-7 (ASIA- C) tetraplegia in 1992 when he was thrown into a swimming pool and hit the bottom of the pool. This patient had been managing his neurogenic bladder by intermittent catheterisation, but he found great difficulty in performing intermittent catheterisation because of redundant prepuce. With overhanging prepuce, this patient required a caregiver to retract the prepuce and expose the urethral meatus. Only then could he perform self-intermittent catheterisations. This patient wished to achieve self-reliance and did not like to be dependent on his caregiver for performing self-intermittent catheterisation. If a caregiver was not available, he was unable to perform intermittent catheterisation. In order to overcome this barrier in carrying out a self-intermittent catheterisation regime, circumcision was considered for this patient. Circumcision was carried out under inhalational anaesthesia. Histology of prepuce revealed no significant inflammation. There was no significant histological abnormality. Following circumcision, the external urethral meatus was readily accessible. This patient could perform self-intermittent catheterisation without being dependent upon a caregiver.

### *Patient's Perspective*

"Circumcision helped me to perform self-intermittent catheterisations without relying on a caregiver."

## Case 5 – Use of Flexible-Tip Catheter for Intermittent Catheterisation along with α-Adrenoceptor–Blocking Drug to Relax Urethral Smooth Muscle Spasm in a Male Paraplegic Child

A British Caucasian boy, born in 2004, sustained complete paraplegia at the T-11 level in 2009. The child's mother was taught to perform intermittent catheterisations with size 8 French LoFric Primo catheters.

In July 2010, the mother of this young boy reported to the spinal unit that she found difficulty in inserting a catheter of size 8 French LoFric Primo per urethra for intermittent catheterisation. However, intermittent catheterisation was successful when a silicone Foley catheter size 10 French was used. The tip of the LoFric Primo catheter was found to be very stiff, whereas the tip of the silicone Foley catheter was softer. The child's mother was advised to perform catheterisations with a catheter, the tip of which was not very stiff. The spinal cord physician explained to the parents that the goal should be catheterisation without causing injury to the urethra. This boy was prescribed doxazosin 500 μg once a day at night; doxazosin would relax the urethral smooth muscle and facilitate insertion of a catheter for intermittent catheterisations. Subsequently, the dose of doxazosin was increased from 500 μg to 1 mg.

Initially, the child was prescribed oxybutynin 2.5 mg twice a day, but he was leaking urine about 2.5 h after catheterisation. Therefore, the dose of oxybutynin was increased to 2.5 mg, four times a day, but the child continued to leak urine between catheterisations. Therefore, modified-release tolterodine 4 mg once a day was prescribed instead of oxybutynin and the child remained completely dry.

### *Comments*

α-Adrenoceptor–blocking drugs have been used as adjunctive therapy to intermittent catheterisation in selected spinal cord injury patients. Five milligrams of terazosin nightly improved detrusor compliance; patients also reported fewer episodes of incontinence and autonomic dysreflexia[5]. A combination therapy consisting of antimuscarinics and an α-blocker is likely to decrease detrusor overactivity as well as detrusor sphincter dyssynergia. A soft-tip catheter will reduce the chances of urethral trauma during catheterisation. In our spinal unit, we offer patients a variety of catheters so that an individual patient and caregivers can choose the most appropriate catheter for intermittent catheterisations.

## Case 6 – Some Health Professionals are Reluctant to Implement Intermittent Catheterisations in a Sexagenarian Tetraplegic Patient

A 66-year-old British Caucasian male was found lying on the floor in his home; he did not know how he ended up on the floor. CT of his head revealed mild cerebral atrophy; there was no evidence of acute infarction or space-occupying lesion. MRI scan of the spinal cord revealed spinal cord compression at C-3/C-4 and C-4/C-5 due to osteophytes and disc. Decompression laminectomy was performed; the patient's neurological status deteriorated immediately after surgery and was prescribed dexamethasone. He had tetraplegia at the C-3 level Frankel grade C.

He had undergone coronary bypass surgery 5 years ago. He was taking metformin 500 mg three times a day for diabetes mellitus. An indwelling urethral catheter drained the neurogenic bladder. Urine microbiology showed growth of coliforms sensitive to gentamicin and Group B *Streptococcus* sensitive to amoxicillin. This patient developed three episodes of symptomatic urine infection during a period of 3 months. It was likely that the indwelling urethral catheter contributed to the development of urine infections. Therefore, he was advised intermittent catheterisation so that the indwelling urinary catheter could be discarded, but health professionals were reluctant to implement an intermittent catheterisation regime in this patient mainly because of his age.

### *Comments*

Furlan and associates[6] showed that nurses working in the acute-care unit held more ageist attitudes than their rehabilitation-nursing counterparts. Among nurses, a higher level of education and experience working in the rehabilitation setting were associated with fewer ageist attitudes. There was a trend for an association between older age and more positive attitudes towards older patients. Practice of ageism has the potential to prejudice service provision and ultimately recovery of patients with spinal cord injury. Perhaps a solution to ageism would be mandatory in-service training of staff on the awareness of ageism. Further, the hazards of indwelling catheters should be highlighted to all health professionals caring for spinal cord injury patients.

## Case 7 – Urethral False Passage after Using Size 12 French Catheter for Intermittent Catheterisation

An 80-year-old British Caucasian male was prescribed warfarin for atrial fibrillation. Subsequently, he developed epidural haematoma. Decompressive laminectomy was performed, but there was no neurological improvement. This patient had developed T-10 paraplegia ASIA-C (American Spinal Injury Association impairment scale category C). This patient was admitted to the spinal unit for rehabilitation. He started managing his bladder by intermittent catheterisation. He used size 12 French Nelaton catheters. A few weeks later, while he was inserting a catheter, there was difficulty and bleeding ensued. Cystoscopy showed a false passage in the bulbar urethra. A size 16 French Foley catheter was inserted over a 0.032-in. guide wire. Since then, this patient continues to manage his bladder by indwelling urethral catheter.

### Comments

Trauma to the urethra while performing intermittent catheterisation is not uncommon. Lindehall and associates[7] observed difficulties with catheterisation in 12 female patients with myelomeningocele, who used noncoated polyvinylchloride catheters for clean intermittent catheterisation for a minimum of 10 years. The problems were solved by temporary use of lubrication or by other minor changes in management. The risk of difficulties at catheterisation doubled with the use of a Ch8 to Ch10 catheter compared to a Ch12 or larger catheter, and doubled during assisted clean intermittent catheterisation compared to clean intermittent self-catheterisation. *These authors concluded that clean intermittent self-catheterisation with large-size catheters were associated with few complications.*

### Case 8 – Difficulty in Performing Intermittent Catheterisation in a Female Patient with Tetraplegia

A 42-year-old woman developed an extensive extradural collection of pus tracking along the whole length of the spine. In the cervical region, the collection of pus was located interiorly, causing compression of the cervical cord from the C-1 to C-3 levels. Decompression of the cervical spine/foramen magnum was performed.

Postoperatively, this patient developed tetraplegia C-5 (ASIA-B). This tetraplegic patient underwent rehabilitation in the spinal unit. During her stay in the spinal unit, she had an indwelling urethral catheter and developed several episodes of serious urine infections. Ultrasound examination revealed normal appearance of both kidneys; there was no hydronephrosis. We were unable to implement intermittent catheterisation in this patient mainly because this patient required hoisting on to a bed for catheterisation. Access to the urethral meatus was almost impossible when she sat up in a wheelchair because of her body build.

### Comments

Joiner and Lindan[8] taught self-intermittent catheterisation to 24 women with neurological diseases involving the bladder. All patients were able to perform the procedure while in the hospital, but for four patients, intermittent catheterisation proved to be impractical and was discontinued after 1–2 weeks. After discharge home, three patients changed to permanent Foley catheter drainage. Women with social problems and those women confined to wheelchairs faced practical difficulties in adopting intermittent catheterisation. Architectural barriers, time schedules, and lack of suitable clothing to enable easy access to the urethral meatus for intermittent catheterisation were the major constraining factors.

## DISCUSSION

Studies have shown that bladder management with an indwelling catheter for patients with spinal cord injury is associated with urological complications, such as stones, urinary infection, urethral strictures, and bladder cancer. Long-term intermittent catheterisation is likely to reduce the risk of these complications in patients with spinal cord injury. An important factor that motivates spinal cord injury patients to accept the hassle of long-term clean intermittent catheterisation is continence[9]. Cameron and associates[10] determined the method of bladder management at discharge from rehabilitation and at each 5-year follow-up period for 30 years from 1972 to 2005 by using the National Spinal Cord Injury Database in the U.S. Individuals using clean intermittent catheterisation at discharge home did not continue to use this method; only 20% of patients who were initially on clean intermittent catheterisation remained on this form of bladder management.

During the year 2010, spinal cord injury patients and physicians at the Northwest Regional Spinal Injuries Centre, Southport, U.K. experienced practical difficulties to successful implementation of an intermittent catheterisation regime. Some patients were able to solve the problem, but the barriers proved impossible to overcome in others under the existing social setup. In order to reduce the complications of long-term indwelling catheter drainage, such as urine infections as indeed experienced by Patient No. 8, spinal cord physicians need to identify the barriers to implementing intermittent catheterisation. Only when specific difficulties encountered by spinal cord injury patients are recognised can remedial measures be taken. Of course, the first step is to raise awareness of the seemingly mundane issues that prevent successful implementation of an intermittent catheterisation regime. This report of eight patients who experienced problems in carrying out intermittent catheterisation is aimed to create such awareness amongst health professionals and policymakers. Hopefully, this will lead to improvement in the facilities for spinal cord injury patients, both in the community as well as in the hospital setup.

Another important aspect is the changing demographic of patients with spinal cord injury. At the Northwest Regional Spinal Injuries Centre in Southport, we are beginning to see this change, especially in the age of patients who are admitted to our unit. Ageing spinal cord injury patients require a change in the attitude of health professionals so that health professionals do not bear a negative attitude to people who are in their seventh or eighth decade of life. This situation is not peculiar to spinal cord injury alone. In the near future, over 40% of patients with lung cancer will be >70 years old at the time their disease is diagnosed[11]. Age per se, however, should not lead to the denial of a potentially curative surgical intervention. It has been shown that older patients (>70 years), as well as patients >80 years of age, may tolerate a lobectomy or even a pneumonectomy quite well.

When intermittent catheterisation through the urethra is difficult, continent catheterisable vesicostomy can be considered. Touma and associates[12] performed Casale Spiral Monti vesicostomy in 12 patients (mean age 27.4 years) who were followed up for a mean of 2.8 years. The results of this study demonstrated that Casale Spiral Monti vesicostomy could have dramatic positive effects on the quality of life in adults with a neurogenic bladder by granting them social independence, convenient bladder management, and excellent continence rates. We performed Benchekroun continent vesicostomy in a female patient with spina bifida and paraplegia to enable her to perform self-catheterisations[13]. This patient developed severe stenosis of the Benchekroun stoma, stones in urinary bladder, and ventral hernia; she required permanent suprapubic cystostomy. This case was a poignant reminder to us that novel surgical techniques should be viewed cautiously and patients should be informed of potential complications of surgical procedures, some of which could be irreversible.

## CONCLUSION

These cases illustrate the need for (1) education and motivation of patients and caregivers to carry out intermittent catheterisation; (2) availability of caregivers or nurses to perform five or six catheterisations a day; (3) provision of toilet facilities in public places and shopping complexes where caregivers can perform intermittent catheterisation on spinal cord injury patients; (4) correct choice of catheter, which is most appropriate for intermittent catheterisation for the individual patient; (5) overcoming mechanical barriers to intermittent catheterisation, such as redundant prepuce; (6) avoiding trauma to the urethra while performing intermittent catheterisation at all times; (7) prescribing α-adrenoceptor–blocking drugs to relax the urethral smooth muscle in spinal cord injury patients with spasm of the urethral sphincter; and (8) encouraging health professionals to make extra efforts to perform intermittent catheterisation in female spinal cord injury patients and in those >60 years.
